# "The Hospital" Nursing Mirror

**Published:** 1900-01-27

**Authors:** 


### "The Hospital" Nursing Mirror.

Being the Nursing Section of "The Hospital."

[Contributions for this Section of "The Hospital" should be addressed to the Editor, The Hospital, 28 & 29, Southampton Street, Strand, London, W.C., and should have the word "Nursing" plainly written in left-hand top corner of the envelope.]

### Notes on News from the Nursing World.

### THE PRINCESS OF WALES'S NURSES LEAVE FOR SOUTH AFRICA.

Among the passengers on board the "Kinfauns Castle," which had such a splendid send off on Saturday from Southampton, were six of the Princess of Wales's nurses for the war, namely, Miss L. E. V. Asman, Miss M. I. Burdett, Miss A. E. Davidson, Miss M. F. Lightfoot, Miss L. B. Peers, and Miss J. E. Skillman. The Princess of Wales, with characteristic thoughtfulness, sent from Sandringham a large case, containing a down pillow for each of her nurses. As we have heard from several sources that there is a great dearth of comfortable pillows amongst the workers in South Africa, the additional gift of the Princess will be all the more appreciated. The other nurses on the vessel included Superintendent Nursing Sister S. E. Oram, who has been attached to the Army Nursing Service since May, 1886. Miss Oram, who was trained at the London Hospital, was the recipient in 1896 of the Royal Red Cross. There were also on board Nursing Sisters A. L. Wilson, R. Lawles, Jones, Creighton, and Teape. The "Kinfauns Castle" left Malta on Wednesday.

### NURSING CONVALESCENT SOLDIERS IN CONVALESCENT HOMES.

In consequence of the number of inquiries respecting the nursing of convalescent soldiers in convalescent homes we applied for authoritative information to the general secretary of the Soldiers and Sailors' Help Society, in whose hands the arrangements are placed by the War Office. We learn officially from Major Malet, however, that the society do not intend to employ nurses at all, as only such soldiers as are capable of looking after themselves will be sent to the homes for the use of convalescents. A certain number of beds in existing convalescent homes have been put at the disposal of the society, and in these the more severe cases which may require the occasional assistance of a nurse will be accommodated, the nursing being done in each instance by the existing staff. It will probably be three months before any soldiers are transferred from hospitals to convalescent homes, and Major Malet anticipates that most of the men will then mainly require plenty of rest, fresh air, and good food to complete their cure.

### THE NURSES FOR THE BASE HOSPITALS.

As both the Imperial Yeomanry hospital and the hospital to be equipped at the expense of Mr. Alfred Mosely, of Hadley Wood, are to be base hospitals, female nurses will be employed. The strength of the staff in the former will be 30 nurses, and in the latter, which will contain 100 beds, there will be five. To save disappointment it may be stated that only nurses belonging to the Army Nursing Service or the Reserve will be allowed to take duty, but it seems probable that it will be found necessary to increase the Reserve from outside.

### THE LATE MISS M. C. ROSE.

The reality of the risks to which nurses in South Africa are exposed is attested in the sad case of Miss M. C. Rose, who went out recently to the seat of war. Her death at Durban from dysentery has caused a painful shock to a number of nurses who knew and highly esteemed her. She was sister at the Guards' Hospital, Rochester Row, at the time she was selected for service among the sick and wounded, and left England full of hope, eager to devote her best energies to those who most needed them.

### MISS E. RYAN'S CAREER.

The new Royal Red Cross Nurse, Miss E. Ryan, was trained at the Westminster Hospital for three years. At the end of her training she obtained the post of charge nurse at the Memorial Hospital, Kendal. Leaving Westmoreland for the Victoria Hospital, Netley, she entered the Army Nursing Service in 1885, and a year later was ordered to the Citadel Hospital, Cairo. In 1887 she was transferred to Malta, and remained there until 1891, when she returned to England on her promotion to the rank of superintendent. She was next sent to the Military Hospital, Devonport, then, in 1895, to the Cambridge Hospital, Aldershot, and, finally, in January, 1897, to the Station Hospital, Valletta.

### ST. GEORGE'S INFIRMARY, FULHAM ROAD.—A COMMITTEE MEETING SUMMONED.

A meeting of the Visiting Committee of St. George's Infirmary, Fulham Road, has been summoned for Monday afternoon at which, we presume, the charges against the parish authorities of St. George's, Hanover Square, will be discussed. Meanwhile, we have received, and give publicity elsewhere to, several letters written by independent people who are fully acquainted with the position. "A Visitor" conclusively proves that "everything" is not done for the patients' comfort, by the statement that new bread is invariably given in the women's ward, notwithstanding that several elderly patients suffer from indigestion. "Scrutator," it will be observed, says that three of the very abuses to which attention was drawn in the "Mirror," have been known for some time to him, namely, the insufficient food supplied to patients; the inadequate number of nurses; and the presence of boys in the wards which should be confined to men. He hopes that "immediate steps" will be taken to bring the infirmary more on a level with similar establishments in the metropolis. We hope so, too; though the parish authorities of St. George's, Hanover Square, with their immense resources, should not follow, but should lead the way in the matter of necessary reforms.

### THE SALARIES OF NURSES IN INDIAN HOSPITALS.

In consequence of the difficulty of obtaining nurses—a difficulty which, by the way, does much to account for the number of nurses sent out from England to undertake plague duty—the authorities of the General Hospital at Madras decided to take measures to attract recruits. A strange proposal was made by the matron-superintendent and endorsed by the medical officer. It was that the head nurses in the hospital merely should have their pay further increased, the idea being that the step would tempt young women to offer themselves for service in the hope of rising to the higher posts. This scheme was not endorsed by the Indian Government, who, more wisely, resolved to increase the pay of the whole nursing staff, which will in future range from 35 to 70 rupees a month, with the addition of 20 rupees as ration allowance. Previously, the salaries in Madras did not go beyond 40 rupees a month, so that a very important change has really been made. We do not understand, however, why the advantages conceded at the Bombay hospitals, where a nurse is allowed to earn a considerable sum from private cases, and is provided with a uniform, should be denied in Madras.

### A GOOD EXAMPLE BY RAILWAY SERVANTS.

The Barry Nursing Association is indebted for a substantial increase in its income to the railway servants. The men were invited by the committee to contribute to the association, with the result that 563 consented to give a penny a week towards it, 170 refused to assist, and 12 intimated their intention of subscribing annually a lump sum of 4s. 4d. This means an addition of £110 a year, or, in all, an income of £300 a year in regular contributions from the working men of the town. That sum represents half the expenditure of the association, and is an exceedingly creditable proportion. Why should not the substantial support given by the working men of Barry be imitated all over the country? The nursing associations exist almost exclusively for the benefit of a particular class; and, though it is both a privilege and a pleasure to many who do not belong to it to render assistance, there can be no question as to the obligation of those who derive the advantage. In recognising what is due from them, the men of Barry have not only set a good example, but ensured additional help from other people to the Nursing Association.

### MORE NURSES WANTED AT COVENTRY.

We are not surprised that the chairman of the Coventry and District Nursing Institution considers four nurses in a town with a population of between 60,000 and 70,000 altogether inadequate for the work to be done. The number is all the more unsatisfactory because in Coventry there are many large manufacturers, and the artisans are earning high wages. Colonel Adams suggests the initiation of a system of weekly contributions, similar to that which has been so successfully adopted at Barry. It can hardly be affirmed that the Coventry Guardians fitly recognise the operations of the Nursing Institution, for their annual subscription is but 12 guineas. The fact that the nurses paid 16,695 visits last year, and that an inspector from the Queen's Jubilee Institute reports most favourably as to the management, shows that it only remains for the Coventry people, especially the workpeople, to do their duty.

### THE SCARBOROUGH GUARDIANS AND THE SUPERINTENDENT NURSE.

It will be recollected that some time ago the superintendent nurse at the Scarborough Workhouse Infirmary applied to the Board of Guardians to have the staff increased from four to five, and that, as the Board declined, the entire staff resigned. The House Committee have just had to discharge the duty of choosing a new superintendent. Three applicants were interviewed, and while satisfied with the hospital, each expressed her opinion that five nurses were required. According to the *Leeds Mercury*:—

"Miss Hamilton was appointed by a large majority, but on being asked if she accepted office, told the Board she would like to have a week to consider the matter. Pressed to give her answer at once, she thanked the Board, but declined the appointment. The Board then selected Mrs. Ellis, but on being called in she, too, declined the appointment on the ground that the work would be too heavy with the present staff, and eventually the Board called in the remaining candidate on the list (Miss Lench), and the Chairman (Sir Charles Legard) inquired whether, in the event of her being appointed, she would be willing to accept the situation. She expressed her willingness to do so, and was thereupon appointed at a salary of £40 per annum and 8s. per week in lieu of rations."

For the moment, therefore, the difficulties of the Scarborough Guardians are at an end. But as Miss Lench, who hails from Middlesborough, will probably find that the work cannot be done properly with less than five nurses, there may soon be another crisis.

### A STRIKING CONTRAST.

There is, it seems, a difference, even in England, as to the measure of respect which the uniform of a nurse commands. A nurse, who is at present engaged in Sheffield, informs us that her uniform in that city is a protection. If she goes amongst the roughest of Yorkshire working men wearing it they treat her with deference, and on no occasion has she had to complain of insult on the part of even the idlers about the streets. At the railway station the porters always select her box before that of other passengers, never looking for a tip, appearing to regard a nurse as a personal friend. Her experience is that a very different state of things exists in Liverpool. There she did not care to appear in the streets in any save her ordinary dress, because she found, by the way she was occasionally addressed by men of a certain class, that women of dubious character frequently masqueraded in a nurse's costume. The state of things to which she refers cannot be compulsorily stopped, but we are glad to think that it is not general in all our large towns.

### AN INCIDENT OF NIGHT NURSING IN PALESTINE.

Night work in an Eastern hospital consists of more than actual nursing. One has to be ready for many emergencies. The weather is the night nurse's *bête noir*. On fine nights all goes fairly well, but on stormy nights shutters bang, lights blow out, patients scream, and the native servants—who do not sleep in the hospital, and whose only clocks are the sun, moon, and stars—arrive at all hours, thinking that it is time to begin the day's work. Only the other night, writes a nurse in Palestine, "I was startled by a great bang at one of the back doors, and upon going to see who it was discovered one of the servants, Letethe (a handsome Arab girl) in a great state of anguish. She said she thought she had overslept herself, and that it was breakfast time. I explained that the hour was just three a.m., which astonished her not a little, as she declared that the moon told her it was much later! However, she was convinced at last, and glad to lie down on a couch, where she slept soundly till six a.m. A few days afterwards the hospital cook, Malachi, arrived at half-past four a.m., in a heavy storm. She, too, had mistaken the time. I subsequently heard that the reason Letethe arrived in the middle of the night was partly because she could not sleep on account of worry. Poor little soul—her parents want her to marry a man she hates, and the thought of it kept her awake till in desperation she flew to the hospital for comfort, though she herself is far too proud to own to us the real cause."

### COLCHESTER GUARDIANS AND THEIR "PLEASANTRIES."

At a meeting of the Colchester Guardians last week the subject of advertising for a superintendent nurse was under discussion. It appears that two of the late superintendent nurses married widowers who were members of the Board, and when the clerk suggested advertising for a superintendent nurse Mr. Cant remarked that "an intimation to the effect that there were still a few widowers left should be included in the advertisement"—whereat there was much laughter. It is not long ago since similar remarks attended the appointment of a nurse, the same guardian advising that the nurse should be told as an inducement that "the nurses usually marry from the workhouse." Of course, these brilliant sallies are reported in all the local newspapers, and they are naturally a source of great annoyance to the nurses. But it is not the nurses who suffer in reputation.

### BUXTON NURSING ASSOCIATION.

The record of work done for the year by the Buxton nurses is admirable. Nearly four thousand visits were paid by the two nurses, whose services all the speakers at the annual meeting acknowledged had been of the most satisfactory character. But the financial support given to the Nursing Association is not what it ought to be in a town of such importance as Buxton. The subscriptions and donations only reached £117 3s. The war fund was mentioned, but, as Mr. Rew reminded the meeting, war was always going on in their midst, and it was their duty to do their part, for the war they had perpetually to carry on was with sickness, pain, sorrow and distress. It seems that at least an addition of £50 a year is required. There should not, in the circumstances, be the slightest difficulty in obtaining it.

### THE HARTLEPOOL GUARDIANS AND THE NURSES.

The Hartlepool Guardians, at a recent meeting, decided to ask one of their probationers, who will complete her training in May, to undertake the duties of charge nurse at the munificent salary of £18 a year. We are always hearing the bitter complaint of the scarcity of nurses for Poor Law work, while at the same time salaries are offered for trained nurses which would not tempt a capable parlour-maid. At the meeting in question it was resolved to ask another of the nurses to fill the post of senior nurse, left vacant by resignation, for the liberal sum of £27 per annum. Unless the Hartlepool Guardians make up in dietary and comfort of nurses' apartments what is lacking in salary, they must expect to pay the difference, and more than the difference, in advertising for help.

### FOUR YEARS' TRAINING.

From "Statistical Information from Sixty Hospitals," obtained by the general superintendent and secretary of the Royal Albert Edward Infirmary and Dispensary, Wigan, we notice that the system of four years' training adopted at the Royal Free Hospital, Gray's Inn Road, is now in force at the following provincial institutions:—Glasgow Western Infirmary, Royal Devon and Exeter Hospital, Liverpool Northern Hospital, and Royal Hants County Hospital. In every case the answer given to the question, "Is a certificate given?" is "Yes," but the tests of efficiency vary. To the question, "Are pensions given or assisted to?" the answer is generally "No."

### "AN OLD UNION HAND."

An amusing incident happened to a nurse recently in a provincial union where there is no resident medical officer. Late one evening the superintendent nurse was sent for to see a woman just admitted who, according to her own account, was seriously ill. Having examined her the nurse found no symptoms serious enough to necessitate her instant removal to the infirmary, and it was therefore intimated to the woman that she would stay in the receiving ward pending the doctor's visit on the following day. This did not please the old dame, who was an old union hand, and who would have preferred the better dietary and extra comforts of the infirmary wards. After superciliously scanning the nurse from head to foot, she said, with contemptuous emphasis, "And who are you, I should like to know?" "I! Oh, I'm the nurse," replied the superintendent nurse, who was, by the way, of rather a youthful appearance. "A nurse!" she cried with a sniff; "I suppose that's cos yer run abart in a bloo frock givin' orders. A young thing like you ain't no more fit ter be a nuss nor I'm fit ter stop 'ere all night; anybody can see with 'arf a eye as I'm as porely as porely can be, and a chit of a gel like you ain't no guarantee for the beatin' of *my* pults; but I'll write to Pore Lawr and let 'em know yer carryin's on, yer funny thing, yer." At this juncture the door was gently but firmly closed on the irate and voluble lady, and the nurse went her way with a twinkle in her eye that wasn't there before.

### SHORT ITEMS.

The Duchess of Buccleuch, who presided at the annual meeting of the Dalkeith District Nursing Association the other night, congratulated the society upon the admirable work done by Nurse Burnett, to whose excellent and cheerful nursing, and bright and sympathetic manner, she and others bore testimony.—The people of Hyde have resolved to raise a fund sufficient to maintain a district nurse. The sum required is £100 a year, towards which one lady contributes £50.—The Committee of the Sunderland Nursing Institute were able at the end of 1899 to give bonuses varying from £3 to £7. During the year Nurses Barbara McLaren, Gertrude Leech, Edith Hall, and Minnie G. Fernley were presented with the institute's silver medal of merit for length of and faithful service.—At the annual meeting of the Perth Nursing Society, the Dean of St. Andrews, in moving the adoption of the report, said "it was a wonderful thing to think that something like 8,000 visits had been paid during the year to those who were poor and sick," and he "ventured to add that the true female clergy were the sick nurses of the society."—A new nursing home is to be erected at Queen's Park, Glasgow.—The work of district nursing at Wells during the past year has not been quite so heavy as previous years, but Nurse Smith has been kept fairly busy, having attended 240 cases, and paid 3,741 visits.—An entertainment of an excellent character was provided for the amusement of the patients of the Cancer Hospital, Fulham Road, on Thursday evening, by the Plowden Bijou Orchestra, the various songs and parts being well sustained, and received with much applause.

### Lectures to Nurses.

By Carstairs Douglas, M.D., B.Sc.Edin., Professor of Medical Jurisprudence, Anderson's College Medical School, and Dispensary Physician, Samaritan Hospital, Glasgow.

### DIETETICS AND THE PHYSIOLOGY OF DIGESTION —(*continued*).

Having now completed our survey of the general anatomy and physiology of the gastro-intestinal canal, let us direct our attention to the various food-stuffs, with the digestion and absorption of which this canal is concerned.

All foods may be classified under a few great heads, and a perfect and complete dietary is composed of elements selected from each of those divisions. There is one perfect food, an analysis of which enables us to say what are the essentials of a complete dietary. I refer to milk, which can itself supply the young mammal with all the elements necessary for its growth and development. An analysis of milk reveals that it contains (1) certain substances which are termed proteids; (2) a sugar named lactose, which comes under the heading of carbo-hydrates; (3) fat in the form of cream; (4) salts; (5) water. Therefore we say that a complete physiological diet must be composed of these five classes of food-stuffs. Let us now discuss them in a little more detail.

1. Proteids.—In this group are collected a large number of important food materials, derived from both the vegetable and animal kingdoms. They are sometimes called albuminous foods, since albumin, or white of egg, is a good example of this class, and they are often termed nitrogenous foods because they contain about 15 or 16 per cent. of the element nitrogen. They also contain oxygen, hydrogen, carbon (50-60 per cent.), sulphur, and occasionally phosphorus as well. They are absolutely necessary for the proper performance of all vital functions, since they alone can replace the waste of protoplasm in the body. Among typical examples of proteids may be mentioned myosin from muscle, fibrin from blood-plasma, albumin from white of egg, and serum globulin; these are sometimes called simple proteids. Others are termed compounds, such as the nucleo-proteids (*e.g.*, casein in milk), hæmoglobin (contained in red blood cells), and mucin. A third and subordinate group is named albuminoids, and embraces such bodies as collagen, contained in white connective tissue; gelatin, obtained by boiling collagen; and elastin, in yellow elastic tissue. All these substances are from the animal kingdom; from the vegetable group we get gluten, an important proteid yielded by flour, and legumin, contained in peas and beans.

2. Carbo-hydrates.—In this class we have a number of very important foods, which, however, differ greatly from proteids. In the first place, they contain no nitrogen, being composed only of the three elements—carbon, hydrogen, and oxygen; in the second place, in all of them, the hydrogen and the oxygen occur in exactly the same proportions as they do in water, viz., two volumes of hydrogen to one of oxygen; and thirdly, all the carbo-hydrates in our food, except the sugar in milk, are supplied from the vegetable kingdom. They may be divided into three sub-groups: (1) The glucoses, containing dextrose or grape sugar, and levulose; their chemical formula is $C_6 H_{12} O_6$; (2) the sucroses or saccharoses, comprising cane sugar, lactose or milk sugar, and maltose or malt sugar; their formula is $C_{12} H_{22} O_{11}$. (3) The last sub-group contains the amyloses, of which by far the most important member is starch; other members of this group are dextrin (a kind of gum), glycogen or animal starch, and cellulose, the substance out of which the woody fibres of plants are built up; the formula of the amyloses is some multiple of $C_6 H_{10} O_5$, expressed usually $(C_6 H_{10} O_5)_n$.

3. Fats.—In this class we have a number of bodies of an entirely new chemical composition. Sometimes the name hydro-carbon is given of members of this class, but this is wrong, since hydro-carbons are bodies composed only of carbon and hydrogen, such as marsh gas. Fats, however, are derived from hydro-carbons, but contain oxygen also, and one of the peculiarities about fats is, that the oxygen is present in much smaller proportion than the two other elements; fats are, therefore, as it were, in an unsatisfied state with regard to oxygen, and accordingly readily take it up if it is presented to them. This union with oxygen takes place in the body, and since oxidation is accompanied by heat, we find that the fats are the foods *par excellence* which maintain the body heat. In their chemical constitution fats are compounds of a fatty acid and glycerin, and every fat can be decomposed to yield these two components; this, indeed, is one of the changes which fats undergo during pancreatic digestion. Typical examples of fats in food are palmtin and stearin, which are solid at ordinary temperatures, and olein, a fluid fat. Most of our fats are obtained from the animal kingdom, *e.g.*, the fat of beef and mutton, and the cream of milk; but some, such as olive oil, are yielded by plants.

4. Salts.—In practically all the fluids and solid tissues of the body we find a certain amount of salts, of which the most important are the chlorides of sodium and potassium, and the phosphates of lime and magnesia. Iron also may be included in this group for convenience, though we usually ingest it in an organic form. In general, sufficient amounts of the various salts for the needs of the body are found in our ordinary food; in the case of sodium chloride, however, or common salt, so much is required that we are in the habit of taking a certain amount every day with our food, in addition to that contained in it.

5. Water.—This, the last of our materials, is also absolutely essential for the balance of health and the maintenance of the vital functions. As a matter of fact, persons threatened with starvation can subsist much more easily without solid food than without water. It is only necessary to state that more than half of the body-weight consists of water, in order to make clear how needful this element is in our daily food. As regards the special part played in nutrition by the chief groups of food stuffs, it may be noted: (1) That proteids are necessary for the development and repair of the nitrogenous tissues of the body, for the elaboration of bodily fluids and secretions containing nitrogen, and for the functional activity of the body generally; (2), that fats act partly as "proteid-sparers," that is to say, they save nitrogenous waste in the body, and by their presence in a dietary render a great amount of proteid food unnecessary; they further, through oxidation, yield us a great deal of muscular energy and heat, and they also enter into the structure of the bodily tissues; (3), the carbo-hydrates act in much the same way as fats, but they do not help to build up the bodily tissues as proteid and fatty foods do; they are especially valuable as sources of energy and heat from their combustion within the organism.

We have now completed our survey of the great classes of food-stuffs; let us study in some detail the digestive changes which the latter undergo, in order that they may be fitted for absorption and assimilation. Let us suppose that a person has taken a meal composed of beef, of bread and butter, seasoned with a little table salt, and washed down with a tumbler of milk. In the mouth the ptyalin of the saliva acts upon the starch in the bread, but not upon proteid or fatty foods. In order that ptyalin may act on starch the latter must first be cooked, that the ferment may penetrate within the burst envelope of the starch-grain.

(*To be continued.*)

### Nursing at the Military Hospital, Malta.

By a Visitor.

The nursing in the Military Hospital, Valletta, Malta, whose lady superintendent has just been decorated by the Queen with the Royal Red Cross, is conducted by a staff of Army Nursing Sisters, consisting of one superintendent and two nursing sisters. They are assisted by a ward master and orderlies of the Royal Army Medical Corps. The ward master is responsible for the discipline and cleanliness of the ward and its equipment.

The duties of the orderlies are various. They distribute the diets, wash the patients, make their beds, and discharge many other nursing duties. There is a special ward, which contains fifty beds, twenty-five on each side of the ward. In this ward are placed all the serious cases. Most of these are cases of fever, or Mediterranean fever, as it is usually designated. There are two nursing sisters, one in charge of a side of twenty-five beds, and from four to six orderlies and a ward master. The ward is never left, either by day or by night, without attendants, who take their "tour of duty" every two or three hours at a time. One orderly is in charge of the ward equipment.

### The Duties of the Nurses.

The superintendent and nursing sisters are responsible for the nursing of the patients under the medical officer. They take the temperature, serve the medicine and the stimulants, and see that all the treatment is properly carried out according to instructions given to them. When there are spinal cases a sister visits the ward at night, and, before going off duty, each sister writes a night report, with instructions as to feeding helpless patients, serving medicine and stimulants, and other important matters which might be necessary to bring to the notice of the night orderlies. Where there is no regular night sister the ward master sees that these instructions are carried out. But at stations where there is a staff of three nursing sisters and one superintendent, in the event of severe cases, each nursing sister takes her turn of night duty for one week at a time. The hospital holds 200 beds. In the wards where there are no nursing sisters the orderlies carry out all the treatment, the ward master being responsible under the medical officer. The ward master in some ways really supplies the place of a ward nurse.

### Mediterranean Fever.

Although Mediterranean fever is always about more or less, the summer is the time when it is at its meridian, from about June to October. In severe cases of prolonged fever change of air to England is considered absolutely necessary, for when the patient's temperature reaches from 104 to 105 for a considerable time, his condition when approaching towards convalescence is so exhausted, and so invariably accompanied by rheumatism, that nothing but his native air seems to put any life into him again. As a rule very few English people escape an attack of fever, either in a mild form or otherwise. If they do steer clear of an attack while they are in Malta they invariably get a severe attack on returning to England after being there about a month or six weeks. This is unaccountable, but true nevertheless; there are several instances of it on record.

### Febricula Fever.

The fever, which only lasts from eight to ten days, and sometimes not so long, was at one time called "Febricula," to distinguish it from the severe form; this term is obsolete. The terms now used are Mediterranean fever and simple continued fever. The Mediterranean fever goes on for months at a time, with intervals of normal temperature sometimes for a week or a fortnight, and for no apparent reason runs up again to 103 or 104 deg. Fahr., and a severe relapse sets in. Very often several of these relapses occur, which make the fever most tedious and wearisome and exhausting to the patient. The simple continued fever resembles the Mediterranean fever symptoms at the beginning—namely, high temperature, thirst, pains in the back and head; but when the temperature becomes normal it remains so. The individual then picks up strength and is soon quite well again. The symptoms resemble those of influenza in some instances.

### The Malta Season.

The winter months in Malta are very pleasant from November to May, before the great heat sets in, and when the worst "sirocco" is over. It is at its worst in September and October, and on that account those months are the most trying of the whole year. The damp heat, which lasts both day and night, makes one feel in a kind of vapour bath, and a very hot one, too. But from November to May there is bright sunshine every day, with few exceptions, and if it rains in the morning it clears up in the afternoon. February is the coldest month in the year, but the cold is not severe. The wind at times is unpleasant, and the harbour cannot be crossed when the "Gregalier" is blowing. This wind usually lasts for three days, and causes much inconvenience to those who are obliged to get from one point to another. There is much of interest in Malta to the casual visitor, and it seems a favourite winter resort for English people. It is, as a rule, a gay and fashionable spot during these months, owing probably to its being such an important and large naval and military station. There is a very good-sized opera house and also the Manael Theatre.

### Hints to Visitors.

Visitors coming to Malta for the winter would do well to provide themselves with a good umbrella and a few wraps. A large supply of warm clothing is not necessary, and the fish moth is most destructive, especially when it can have an opportunity of transferring its affections to woollen garments. This moth makes its way into almost everything—silk, paper, and even pictures do not escape it. Light silk material or beige is useful. Everything in the way of dress fabrics or millinery is much more expensive than it is at home. Neither are the fashions quite up to date. Visitors generally bring the latest fashions with them. Vegetables are cheap and plentiful; also fruit, and a variety which can be had all the year round at a very moderate price. Oranges last from November to May, and in the summer months there are two kinds of melons. There are also loquats, grapes, and strawberries, which have a very good flavour but are very small, resembling the wild strawberries in England.

### To Nurses.

We invite contributions from any of our readers, and shall be glad to pay for "Notes on News from the Nursing World," or for articles describing nursing experiences, or dealing with any nursing question from an original point of view. The minimum payment for contributions is 5s., but we welcome interesting contributions of a column, or a page, in length. It may be added that notices of entertainments, presentations, and deaths are not paid for, but, of course, we are always glad to receive them. All rejected manuscripts are returned in due course, and all payments for manuscripts used are made as early as possible at the beginning of each quarter.

### New Year's Eve at Arezzo.

By a Travelling Correspondent.

It was the last day of the old year, and even the sick and sorry in the hospital—those who could neither dance nor sing—were looking forward to the festa to-morrow. Patients of all sorts—medical, surgical, mental, incurable, and maternity—were gathered together under the wide-spreading roof of the huge stone building. The convalescents were strolling freely about the corridors, looking into the various wards and exchanging remarks and jests with the attendants and patients. A continual clatter of voices rose and fell in an uneven cascade of sound. When the voices lulled for an instant there was heard the shout of some madman in the mental wards or the faint cry of a new-born child from the wing set apart for the maternity cases.

Several of the convalescents were standing within the door of one of the medical wards talking to the young contadino on the nearest bed. He was a broad-shouldered, brown-faced youth, and his dark eyes looked hungrily out from under his brows. He was just recovering from typhoid, and his friends were condoling with him.

"Sister won't let you eat anything, Virgilio, even should the grandmother bring you fritters to-morrow?" said one inquiringly.

"Che disgrazia!" muttered the lad. "And I could eat an ox without salt—in its skin for that matter. How can a man rise up upon milk?"

"My nephew will bring me a fiasco of wine of Montpulciano to-morrow—no more milk for me," said a lively man with a grey beard.

The others looked at him enviously. The wine of Montpulciano was like velvet on the tongue; a whole flask of nectar ought not to be bestowed on one man. They threw cold water on his anticipations.

"It costs twenty-five centesimi to bring it through the city gate, and your nephew is poor, Giovanni," said one.

"The centesimi will find themselves," said Giovanni, rather uneasily.

"The little Tonina at the fruit-shop will ask him for a drink as he passes by. She has bright eyes, and he will not refuse her," added another.

"A fiasco once broken is soon dead," said the rest in chorus.

The sister sitting at her desk just inside the ward thought they were making too much noise and sent them away down the corridor. At the end was the barred iron door leading into the mental wards, and they stopped there watching the restless movements of those within.

Virgilio left alone felt more hungry than ever; he would have gnawed his bedstead had it been of wood instead of iron. He took a piece of the linen sheet between his white teeth and would have bitten it to pieces if he had not caught the quiet eye of the sister. She left her desk, took the linen from his mouth, tucked him up tidily, and told him to pray for patience.

She left him apparently soothed, and went on some errand to another ward. The lad watched her go out of the door, and before the skirt of her blue gown had disappeared gave a tug that threw half the bedclothes on the floor. After that he lay still and wondered why there seemed so little strength in him—he who could carry a half-grown calf up the hill. If they would only give him some food. He began to shed great tears of sheer weakness.

A lad came into the ward carrying a parcel. Not finding the sister he laid his burden on the desk and went away. The shape of the parcel caught Virgilio's tearful eye; a package familiar enough at New Year and Easter, flat-bottomed, and raised in the centre. The poor fellow trembled as he guessed its contents—a panetone, a large bread cake with currants, was wrapped in the paper.

He looked furtively round the ward; most of the patients were lying with closed eyes; at the farther end two lads were chatting, one leaning out of bed to make his voice reach the other. Old Beppo in the centre bed was sitting propped up with pillows, but Beppo could scarcely see beyond the tip of his nose. No one had noticed the parcel. Virgilio made an effort and raised himself; he stretched one weak leg out of bed and reached the floor. Then the world went round with him, and his pulses throbbed through all his body like giant hammers. Still he was not beaten; the desk was near, and he let himself slide from bed to floor. He took another unsteady glance down the ward, but no one was looking; then he crawled along on the bricks, reached for the panetone, and with a violent effort got back to bed with his treasure. He pushed it under the clothes just in time—a moment later the sister entered the ward.

He lay quite still, every nerve trembling, and the perspiration oozing from his dark skin. If he had had any strength remaining he would have crushed the big cake flat under his body, but he was utterly exhausted. He could only lie still and wonder if the sister would notice that curious swelling under the bed-clothes. Presently he would tear the paper and break off some crumbs—when the lights were out and everything was quiet. Madonna give him patience to wait till then!

More parcels arrived, and were piled on the sister's desk. There were dried fruit, confetti, roast chickens, and flasks of wine; panetoni, large and small, and other cakes without number. The sister was very busy reading the names, and deciding whether the patients to whom the gifts were sent were well enough to receive them. She unrolled a large sausage odorous with garlic. "Numero 6—enteric fever," she said, putting it onone side. Poor No. 6 would have no present on the morrow.

The *infirmière* came in and began to tidy the ward for the night; the sister left her desk to superintend. Virgilio had forgotten that his bed would have to be made comfortable and the sheets straightened. An agony of despair seized him, he tried to roll over on to the panetone, but was powerless to move. The sister's eye, travelling from bed to bed, rested for one long second on the lump under the quilt. She came quietly across the ward, uncovered the packet, and took it back to the desk, placing it among the other gifts. Virgilio lay speechless with closed eyes, but great salt tears forced themselves under the dark lashes and fell one by one on the pillow.

### Minor Appointments.

Fulham Infirmary.—Miss Clarissa Mary Clark has been appointed Sister. She was trained at St. Marylebone Infirmary, and was subsequently a staff ward nurse. Since November, 1896, she has been a member of the Nurses' Co-operation.

Stockport Isolation Hospital.—Miss A. Hughes has been appointed Charge Nurse. She was trained at Worcester General Infirmary, and has since been staff nurse at East Dulwich Infirmary; charge nurse at St. Pancras Infirmary, Dartmouth Park Hill; and charge nurse at the Isolation Hospital, Worcester.

### Papers for Probationers.

### TAKING THE RESPIRATION.

To take a patient's respiration, lay your open hand upon his chest, just below the lower end of the sternum or breast-bone, and count how many times he breathes in a minute. This is not always an easy matter, as the respiration, being more or less under the patient's control, often alters, consciously or unconsciously, when he knows he is being observed. In such cases one can often count the respiration by watching the rise and fall of the chest while still apparently taking the pulse. With some neurotic patients the normal rate can only be ascertained during sleep. I remember one old hypochondriac who puffed away like a steam engine whenever he thought his breathing was being observed, getting in from 40 to 50 respirations per minute, but when he was asleep they dropped to the normal number, from 16 to 20.

In diseases affecting the pleuræ and lungs the respirations are increased out of proportion to the temperature and pulse. A rise of temperature is nearly always accompanied by increased frequency of respiration. In conditions of collapse, of profound unconsciousness from concussion, alcoholic or chloral poisoning, &c., the respirations, on the contrary, are slow and shallow.

The same remarks as to the effect of violent exertion, excitement, &c., on the pulse, apply equally to the respiration, and, as with the pulse, the rate is not the only thing to be taken account of by the nurse. What effect the act of respiration has on the patient, whether the breathing is influenced by position, what are its character and sound, and what the movements of the chest, are all points the nurse must observe.

### How the Patient is Affected.

Is breathing accompanied by pain, is there difficulty in breathing, or is there mere "shortness of breath"? Perhaps the patient complains of a sudden pain like a stab each time he draws his breath, and often can put his finger on the exact spot at which he feels it. You may expect to find this pain in cases of fractured ribs, acute pneumonia, and pleurisy. In the first case it is due to injury or pressure of the lung or pleura. In the latter the pain is caused by the rubbing over each other of the surfaces of the pleura roughened by inflammation. This rubbing is called "friction." When the pleurisy is accompanied by fluid between the pleural walls the pain is relieved; the friction ceases, as the roughened surfaces no longer come in contact, but are separated by the fluid —"pleurisy with effusion." Though this condition gives rise to less absolute pain, the difficulty of breathing is increased, as the pressure of the fluid prevents the lung expanding freely. A large effusion will sometimes displace the heart, pushing it against the sound lung, and so embarrassing its action as well. When the fluid takes the form of pus the patient is said to have an empyema. Pneumonia is generally accompanied by pleurisy, and thus the same pain is felt on breathing. When the patient cannot get rid of the air in his lungs, and the expiratory movements are slight, the lungs having lost their elasticity and being clogged with air, he is said to be suffering from emphysema. Do not confound the two terms empyema and emphysema. Dyspnœa, or difficult breathing, may be due to various causes, such as the pressure of a tumour, obstruction of the trachea or larynx, and, commonest of all, to heart disease, when the lungs become engorged with blood from defective circulation. Sometimes the dyspnœa is so severe as to be an immediate danger; the patient's face and ears are first livid and then become almost black, his eyes appear to be starting out of his head, the great vessels of the neck stand out throbbing and swollen, and the patient can only make short gasps. No time must be lost in reporting such a condition, and the patient must be propped up and any tight bandage or garment loosened from the neck or chest.

### Position.

Notice in what position the patient breathes with greatest ease. In pleurisy, pleurisy with effusion, pneumonia, &c., he will lie on the affected side, to give the other lung free play. If both lungs are involved he will probably lie on his back, and if a large part of the lungs are useless he will beg to be lifted and propped up in bed. Sometimes the patient cannot breathe at all except in an upright position; this condition is called orthopnœa. He will often be greatly relieved if allowed to sit on the edge of the bed with his legs hanging over the side (of course, wrapped in a blanket). Some doctors allow such patients to sit in an arm chair all night instead of going to bed. You can realise the discomfort of being obliged to sit bolt upright in bed with legs extended at a right angle in front of you, and you must never get into the habit of thinking care for your patient's comfort "fussy" or "faddy."

### Movement of the Chest.

This may be slight or even entirely absent over the affected lung. The upper part of the chest only may move, thoracic breathing; the abdominal walls may be sucked in with each inspiration, a very grave symptom.

### Character and Sound.

The respirations may be deep or shallow, quiet, or noisy and stertorous, while the cheeks are puffed out with each breath, as in cerebral hæmorrhage. The stertor is due to paralysis of the soft palate, causing it to hang down and obstruct the free exit and entrance of air to the lungs. Propping the patient on his side frequently gives relief. The rattle of bronchitis, the crowing of croup, and a similar sound produced by other laryngeal or tracheal obstruction, and the brassy cough accompanying a thoracic aneurism, and many other peculiarities you will learn to observe and recognise. Hiccough, which is a spasm of the diaphragm, must never be allowed to pass unnoticed; it is often a serious symptom.

Two important phenomena remain to be described, viz., "air hunger" and "Cheyne-Stokes respiration." Air hunger occurs in a very grave condition known as diabetic coma. It consists of greatly exaggerated movements of inspiration and expiration, as though the patient were gasping for air, the breathing not, however, becoming rapid. Cheyne-Stokes respiration is not confined to any particular disease, but occurs in many serious conditions. It consists of a series of respirations gradually increasing in depth and rapidity till a climax is reached, the respirations then gradually decrease, becoming a mere sigh, when a pause occurs of several seconds' duration. This process may be repeated for hours, or perhaps only for a few minutes. The number of respirations in each series vary from five to fifty or more, and each series may occupy from a quarter to a whole minute. Of course, you will at once report the occurrence of this form of breathing.

### Appointments.

Woolwich and Plumstead Cottage Hospital.—Miss A. D'Arcy has been appointed Matron. She was trained at Sussex County Hospital, and has since been matron at the London Throat Hospital, and matron at the Malmesbury Cottage Hospital, &c.

Lowestoft Sanatorium.—Miss S. M. Jacob has been appointed Matron. She was trained at Sir Patrick Dun's Hospital, Dublin. She has since been head surgical sister in the same institution, and subsequently staff nurse at Darlington Hospital, matron County Meath Infirmary, assistant matron Wolverhampton Fever Hospital, and matron Kendal Sanatorium.

### Echoes from the Outside World.

### AN OPEN LETTER TO A HOSPITAL NURSE.

Before this reaches you the turning-point of the war may have come—and gone! The fear lest we should have failed to drive the Boers from their stronghold of Spion Kop makes one sick with apprehension, because it means so much to us. The suspense inside Ladysmith must, of course, be a thousand times more acute than our own, and there is something quietly pathetic in the heliograph message which came through on Wednesday morning that, though "the rumble of General Buller's guns continues without interruption," the besieged "can trace no development, except that the advance seems to draw the enemy away from the town." In view of the news that Sir Charles Warren has made no move, but only held his own, since last Sunday, it is comforting to remember we were warned that caution would be his watchword, and that he would rather take five days to do his work than run the risk failure. Various correspondents try to reassure us by rumours that the Boers are running short of money, of ammunition, of food, and are tired of the war, but none of these are worthy of much credence. Our chief matter for congratulation lies, as Lord Rosebery put it at Chatham on Tuesday, in the evidence afforded by the conduct of the nation that its ancient courage is as high as ever, and its fortitude as great, and that any apprehensions lest our prosperity should have resulted in moral "fatty degeneration," have proved groundless.

Although it is natural that most of our thoughts should be centred in South Africa, matters are growing too serious in India for any of us to ignore them. Nearly fifty millions of people likely to suffer severely from starvation, if not to die from want of food, sounds almost too gigantic a misfortune for the ordinary mind to grasp, and in thinking of our own trouble we are apt not even to try to do so. But if our possessions in Africa demand much of us, so do our belongings in Asia, and the more, perhaps, because our Indian Empire involves responsibility for so many millions of human beings. Lord Curzon's despairing assertion that he cannot expect England to help just now except with "passive sympathy" is heartrending, and I am convinced that he will find he is mistaken in his judgment. By all means let England give her sympathy, but let it, like silence, be golden. It could easily be done. Some are wealthy and can help liberally, but most of us have strictly limited means. Yet we all can spare two or three pence, and if these were forwarded to the Lord Mayor of Manchester—who is going to open a fund—they would soon bring relief to the stricken natives. Not, possibly, in the generous fashion of 1897, but still in sufficient measure to free us from the reproach in the years to come that whilst we fought the Boers we allowed the natives of our great Dependency to starve.

Death has been busy during the past week. Several well-known men have been taken from us, and the world is the poorer. The Duke of Teck has practically been an invalid since the death of his much-loved wife, and, though a well-known personage at Richmond, is famous to most of us simply because he is the father of the lady who may some day be our Queen. Mr. Ruskin's name, on the contrary, is a household word. Few even of the most busy have not at some time or other read and enjoyed his delightful works. To most it will come as a surprise that his books alone number over seventy volumes, but "Stones of Venice," "Modern Painters," and "Sesame and Lilies" have probably been our bookshelf friends for years, and any inherent love of the best in art which we may have originally possessed has been fanned and fed by the great master. For notwithstanding that much that he wrote, both on artistic subjects and on political economy, is open to controversy, he was a great master, and his place amongst the writers of the reign of Queen Victoria is a very lofty one. Although he never sought to please the public, their appreciation of his works is shown by the fact that the sale of his books, for a long series of years, brought him in an income of £4,000 a year. The girls of Whitelands College, Chelsea, who yearly, with Mr. Ruskin's special approval, select the most lovable of their number to be their May Queen, presenting her with the author's gift of Ruskin's works, will miss the Professor much, and yet, remembering the words of the man himself when writing about the death of a friend, they will hardly dare to mourn. "Oh, dear Susie," he wrote, "why should we wear black for the guests of God?"

It is strange that two such ardent lovers of nature as Ruskin and Blackmore should have passed away so nearly together. The former revelled in the beauties of Coniston; the latter had drunk deeply of the loveliness of the Devonshire moors, and by translating their delights into prose for the benefit of the toilers in the city, as he did in "Lorna Doone," he has given us a heritage for which we shall always be grateful. Young and old, learned or unlearned, all alike revel in the fresh breeziness of his "tale of Exmoor," which he was not able to surpass in any of his subsequent books. Last on the list is Mr. G. W. Steevens, who, after he had appeared to have turned the corner, had a sudden relapse and died of enteric fever at Ladysmith. His articles "With Kitchener to Khartoum" are well known, and all he wrote was eminently readable, owing to his vivid manner of describing details. As a man he was exceedingly liked by all with whom he came in contact, and his death at the early age of thirty is a matter of general regret.

The news that the wily Osman Digna has at last been captured is very welcome, because, although the Mahdi and the Khalifa can trouble us no more, it has always been asserted that as long as Osman Digna was at liberty we should run the risk of little harassing rebellions in a land where otherwise we might have hoped for peace. It is strange that Osman Digna should have possessed so much influence among conspicuously courageous followers, for whenever a big fight took place, which was likely to put his person in danger, he invariably succeeded, with almost marvellous ingenuity, in making himself scarce, and escaped unhurt from El Teb, Handub, Atbara, and Omdurman. Perhaps his constant elusion of the grasp of his pursuers was cited by him as evidence that he was more than an ordinary mortal. Now, however, his power has come to an end, though he does not appear to have been given up by his followers, but to have been simply surrounded and captured. He will probably be assigned an island for his future home, in the same way as Arabi Pasha resides in Ceylon, and care will have to be exercised that he does not escape therefrom; but as yet nothing seems decided upon that point, and for the moment he languishes in a Suez prison.

It is curious how the infant daughter of Sir Francis Wingate has, up to the present, been identified throughout her short life with the important events of the Soudan. She made her appearance in the world the day after her father had succeeded in slaying the Khalifa in battle, and her baptism took place the day before Osman Digna lost his liberty—let us hope, for ever.

### A Book and its Story.

### MR. LEIGHTON'S ESSAYS.

The author of "Life and Letters"* has strung together in essay form some charmingly thoughtful chapters bearing the impress of a critical, highly-cultivated mind. Whether discussing the relative gifts of poets, artists, or prose writers, he is equally suggestive and interesting, and the chapter on Machiavelli, with a new and more generous reading of the literary statesman's character, and that on "Originality" are both valuable contributions.

On Machiavelli, a "political fifteenth century Mephistopheles," the author makes the following comments:—

"I am tempted to think that Machiavelli owes his reputation for diabolical cleverness and intrigue less to his want of morality than to a certain stupidity or obtuseness which led him to criticise, in a highly impartial and philosophic spirit, certain prevalent political methods generally practised in his time but never publicly acknowledged. It may sound paradoxical to suggest that he has earned his reputation by mistaken frankness; but he lived in a benighted political age, when political crime and ruthless treachery were hardly regarded as criminal. . . . He did not invent the special form of politics often called by his name, but he wrote about it, to the great shock of those conscientious rulers of Spain, Italy, and France who did not object to results, but who protested against analysis and revelation of the process. . . . He is eminently practical, ever correcting and verifying his aspirations and theories by the test of human nature, which he regards with philosophic eyes, and his philosophy has deeply impressed him with the transitoriness of all things, not only of individual life, but also of those things which man flatters himself will be permanent, or, at least, long enduring. Machiavelli was one of the first political writers to recognise the political rights of the people at all. He shows a deep sympathy with the wrongs of an oppressed people, and some impatience at their indifference to the tyranny under which they suffered. And this sympathy was direct and sincere." For "Machiavell had none of the savage, gloomy satire of Swift or the gay irony of Disraeli; he is too deadly in earnest to linger over the humorous aspects of life, to be tickled by inequalities and contrasts, and he discusses political problems and their possible solution with an intensity which blinds him to any possible adverse effect on the reader." Of this highly endowed man Mr. Leighton concludes his criticism in the following terms: "He had a cruel and thwarted destiny; everywhere his work was frustrated. . . . In writing he considered it his duty to observe and record without a chorus of praise or blame, but his constant allusions to the corruption of his times might save him from the gratuitous assumption that he opposed them. . . . He watched the swing of the pendulum in the mental as Galileo in the physical world; he saw life, and perceived the significance and connection of seemingly unassociated things, and he anticipated much modern thought." . . . "The genius of a bygone age is not to be judged by his ignorance and mistakes, but rather he should be esteemed for his gifts and appreciate the causes of his deficiencies. But in the case of Machiavelli his genius, his passionate love of freedom and Florence, have been forgotten and his evil reputation accepted without inquiry even in an age of historic research. I cannot say that he is still misread, for I think he has very rarely been read, though he would repay study and stimulate thought."

In "Men's Women and Women's Women" there are some trenchant remarks on the sex from this point of view. The author considers that it is not given to women to realise the personality of the woman a man admires. For women who women fancy men adore, or should adore, and the women they really worship, are very different beings; and a man's ideal man and a woman's ideal man "yield very amusing and interesting results." To Balzac, Thackeray, and George Meredith he gives the palm as the most successful delineators of the siren in various and differing situations. To the question "Should a woman master her husband's work and concern herself fully with it?" the character of Marcella is referred to "as that of a fine-hearted woman tumbling into pitfalls, and increasing rather than lightening, the weary load of public life for her husband." And we confess to leaning to the view "That it would be a depressing future if all wives expected to discuss their husband's work with them instead of amusing them and interesting them in other things."

The interesting chapter on the poetic and artistic faculty, which owes to Mr. Francis Galton its title of "the visualising faculty;" is deeply interesting: "That inward eye which is the bliss (or woe) of solitude, that power by whose means artists reproduce the impressions of form, colour, and sound as they strike the finer nerves, and awaken associations, aspirations, and sad or glad humours." How much the world owes to it! Art Zola defines as "Nature seen through a temperament," and how true the definition is must be obvious to all who are in sympathy with the subject. "The poet is the interpreter in the House Beautiful, pointing out to us the beauty and significance which our duller senses would have missed. . . . External beauty is necessary for sustained and wholly satisfactory inspiration; the arts are dependent on one another, and the neglect of beauty leads to the loss of the poetic impulse, as the intellectual history of Germany for the last three centuries illustrates, when the plastic arts were at their lowest ebb and the physical eye denied the sight of beauty and colour except in form. . . . It is significant that our two finest modern poets, Christina Rossetti and Tennyson, have in their poems built themselves a refuge for their souls from the ugliness of every-day life—the man's a palace of art, the woman's a garden of beauty and life. Perhaps if every-day objects of the outward world had corresponded more nearly to the poetic ideal neither of these had been written."

On the vexed question of "originality" Mr. Leighton has his word. That originality is a faculty, a spontaneous creative mental force, characteristic of genius is, to him, hardly tenable. This view may have been so "previous to the discovery of those wonderful words 'development' and 'evolution,' which account grandly for much we do not understand," but not since, in his opinion. He holds that originality, as a rule, is not the natural characteristic of the uncultured mind, but rather of one in which quick insight or imagination, with a power of rapid comparison or selection, prove the possessor to be both intelligent and cultured. Genius is to him not so much the creative as the adaptive power which enables great men to use a thought they had not first originated; to polish and glorify it, too, by the inseeing light of superior mental vision till it is turned out no longer a half-wrought piece of metal, but valuable coin which shall enrich the world's treasury. There is very much to be said for this view. But whatever a genius may ultimately become, surely his first characteristic is that of spontaneity, which places him always in the van, never in the rear, of his contemporaries in the world of art, science, or literature.

We heartily commend "Life and Letters" to thoughtful readers who prefer something more satisfactory to peruse than the ordinary novel.

* "Life and Letters." By F. F. Leighton. (T. Fisher Unwin. London. 6s.)

### The Princess of Wales's War Fund.

We publish to-day a further list of subscriptions from nurses to the Princess of Wales's War Fund, and remind our readers that the subscription is limited to five shillings. All subscriptions should be addressed to the Manager, Office of The Hospital, 28 and 29, Southampton Street, Strand, London, W.C., and the name and address should always be given. The list will close next week.

Amount already acknowledged, £210 1s. 4d.

| | | | | | |
|---|---|---|---|---|---|
| B. Cutler, P.F. ... | 5 | 0 | E. Fillingham, P.F. ... | 1 | 0 |
| A. Smythe, P.F. ... | 5 | 0 | Nurse Oxborne, P.F.... | 1 | 0 |
| G. Watkins, P.F. ... | 5 | 0 | Nurse Gladys ... ... | 5 | 0 |
| M. M. Beaton, P.F ... | 5 | 0 | Sister Strange ... ... | 3 | 0 |
| "Queen's Nurse" ... | 5 | 0 | Nurse Honnor ... ... | 2 | 0 |
| Nurse Jenny ... ... | 2 | 6 | Policy 5,844, P.F. ... | 2 | 0 |
| E. Boul, P.F. ... ... | 2 | 0 | Nurse Ruth Smith, P.F. | 1 | 0 |
| E. Cunnington... ... | 1 | 0 | A. B. Woodward ... | 1 | 0 |
| E. M. Skinner, P.F. ... | 2 | 6 | R. L. J. ... ... | 12 | 0 |
| L. Bell, P.F. ... ... | 2 | 0 | Three Nurses ... ... | 10 | 0 |
| Nurse Atkinson, P.F... | 2 | 6 | Policy 2,787, P.F. ... | 5 | 0 |
| Cecilia E. Jones ... | 2 | 6 | E. Carment ... .. | 5 | 0 |
| H. Nicholls, P.F. ... | 2 | 6 | M. Shoosmith ... ... | 2 | 6 |
| J. E. Walker, P.F. ... | 2 | 0 | S. Crump ... ... | 2 | 6 |
| E. M. Kelly, P.F. ... | 2 | 0 | A. T., P.F. ... ... | 2 | 0 |
| Absent-minded Giver... | 2 | 0 | Nurse Worrall ... | 2 | 0 |
| L. C. Rippon ... ... | 1 | 0 | E. Hatton, P.F. ... | 4 | 0 |
| Nurse Rodger ... ... | 5 | 0 | F. S. Norton, P.F. ... | 2 | 6 |
| Malmesbury ... ... | 5 | 0 | E. M. Sneyd, P.F. ... | 2 | 0 |
| A. E. Stewart... ... | 2 | 6 | A. T. Kelly, P.F. ... | 2 | 0 |
| L. E. Turner ... ... | 2 | 0 | M. A. Fraser, P.F. ... | 2 | 0 |
| H. M. Parker ... ... | 2 | 0 | J. Connell, P.F. ... | 1 | 0 |
| E. H. Edwards ... | 2 | 0 | | | |

Collected at the Nurses' Tea Party, 27, Brompton Square, January 16th-19th, or Second Collection.

| | | | | | |
|---|---|---|---|---|---|
| Nurse Kate Nelson ... | 5 | 0 | Darkie ... ... ... | 2 | 0 |
| Policy 784 ... ... | 5 | 0 | Mark Antony ... ... | 1 | 0 |
| A Friend ... ... | 2 | 6 | Floskins ... ... | 1 | 0 |
| Nellie James ... ... | 1 | 0 | Diavolo... ... ... | 1 | 0 |
| Cecilia Smith ... ... | 1 | 0 | Balance... ... ... | 0 | 6 |

### Wedding of the late Matron of the Cottage Hospital, Bushey Heath.

On January 15th Miss Violet Müller, second daughter of the late Frederick Müller, of Calton Terrace, Glasgow, and late matron of the Cottage Hospital, Bushey Heath, was married to Thomas Mervyn Wyatt, F.I.C., F.C.S., eldest son of Thomas Henry Wyatt, of Weston Corbett, Hants. The wedding, a pretty and quiet function, took place at SS. Philip and James's, Ilfracombe, the officiating clergyman being the Rev. Hanbury Barnes. The bride, who was married in her travelling gown of violet cloth, was attended by her two sisters in grey, each wearing long gold chains, the gift of the bridegroom. All carried large bouquets of violets. In the unavoidable absence of the bride's uncle, the Rev. Ferdinand Parminter, her eldest sister gave her away. Mr. George Wyatt, brother of the bridegroom, was best man. There were upwards of a hundred costly and useful presents. Before Miss Müller left Bushey she was presented with a silver bowl by the doctors and committee, with silver entrée dishes by the nurses, a case containing silver button-hook and shoe-horn by the maids, silver sugar castors and spoon by the patients; and the wedding gifts included many from old patients and ladies connected with the hospital.

### Presentation.

Grove Hospital, Tooting Grove.—On the occasion of the departure of Dr. F. Foord-Caiger from the Grove Hospital, where he has been acting medical superintendent for the past six months, he was presented by Dr. Rundle, on behalf of the medical staff, the matron and nursing staff, and the domestic staff, with a silver cigarette case, a handsomely chased silver c ke basket, and a silver sugar castor. These gifts were made in token of appreciation of the unfailing courtesy and consideration shown towards the donors by Dr. Foord-Caiger during his temporary residence.

### Everybody's Opinion.

**[Correspondence on all subjects is invited, but we cannot in any way be responsible for the opinions expressed by our correspondents. No communication can be entertained if the name and address of the correspondent is not given, as a guarantee of good faith but not necessarily for publication, or unless one side of the paper only is written on.]**

### ST. GEORGE'S INFIRMARY, FULHAM ROAD.

"A. O." writes: I have been reading your correspondents' statements as to the mismanagement of the St. George's Infirmary. I am glad to see the matter brought forward, as I have been cognizant of most of these facts for some time, and with many others equally interested in the questions raised, would be rejoiced to see these errors remedied. I speak more specially of the necessity of separate wards for children, as constant contact with adult depravity gives them but little chance of developing into respectable citizens.

"K. S." writes: With reference to the remarks in your paper regarding the interior economy and general management of St. George's Infirmary, and in view of what I have independently heard of the case, I think it imperatively necessary that the reforms advocated should be promptly taken in hand, especially as regards a sufficient and reasonable diet being given at all times to the patients, and the practice of placing youths and children of tender years in the wards wherein the lowest and most degraded adults are domiciled. The continuance of the course now pursued can lead to nothing but degradation and eventual crime from the bad language and corrupting conversation which must inevitably tend to the perversion of the morals and future life of the inmates.

"A Visitor" writes: I have read with great interest the articles and letters in your paper concerning St. George's Infirmary, and can corroborate your first correspondent's statements. How can any nurse there say that the patients can ask for anything in reason? It is true she does not say that they get it. One woman asked me as a last favour to get her some dry biscuits. "It's the only thing I fancy." It was, indeed, the last favour, and she did not get the biscuits because I was not allowed to give them to her. "They are full of fancies," I was told, and the doctor will order them if he sees fit. Evidently the doctor was not told of the woman's craving. A good many times I have heard the remark, "Oh, if you only knew what it is to lie here and want something you can eat." Only new bread is given in the women's ward, and many elderly patients who are subject to acute indigestion suffer greatly after eating it.

"Scrutator" writes: I rejoice to see that attention has been drawn in your columns to certain serious defects in the management of St. George's Infirmary. A correspondent has pointed out in your columns three main causes of the inefficiency of that institution, which a little good-will on the part of the committee might easily remove. These are: (1) The insufficient food supplied to the patients, who receive but three meals a day, not always of a very substantial kind; (2) the inadequate number of nurses; and (3) the presence of boys in the men's wards, who are thus subjected to corrupting influences to which the community has no right to expose them. These abuses have been for some time known to me, but not residing within the bounds of St. George I felt a natural hesitation in moving in the matter. If the allegations of your correspondent are untrue it will be easy for the committee to refute them; but if they are well founded, as I believe them to be, they will be incurring a very grave responsibility if they do not take immediate steps to bring their infirmary more on a level with similar establishments in the metropolis.

### LAMBETH INFIRMARY.

"Nurse" writes: I was sorry to see in last week's Hospital that there has been trouble at the Lambeth Infirmary about the ball. Years ago I was a nurse there, when balls and classes were frequent, and, speaking quite impartially, I must say that the matron has not exaggerated the evils that may, and did arise in those days. You may suppose that the nurses were free agents and could do as they liked in the matter—go or stay away; but in my day this was not so. If you had the courage of your convictions then you had to abide by the consequences after, as I knew only too well. I have heard that things are different now, and, for the sake of the nurse who has not been afraid to say what she thinks in last week's HOSPITAL, I rejoice that this is so, and I admire and respect her for her action in this matter.

[* * Our correspondent seems to overlook the fact that Nurse Moloney did not write on her own behalf merely, but on that of the nurses of Lambeth Infirmary generally.—ED. *T. H.*]

### A SPECIAL CORPS OF TRAINED NURSES TO VOLUNTEER FOR THE WAR.

R. V. EDWARDS, writing from Cwmavon House, near Pontypool, says: I endorse every word of the letter by "An Officer's Daughter" in your issue of January 13th, and only hope that if any such corps of volunteer nurses be raised I may be included in it. I am a Guy's certificated nurse, and have had medical and surgical work, both there and since. I would readily pay my own expenses, and I have wondered much that when offers of gratuitous nursing have been accepted from the colonies no proposal of a similar nature has found favour in the old country. It is not for lack of anxiety on the part of the nurses, but for lack of organization, and your own quotation from Mr. Freemantle's letter proves that if such a corps were sent out it would not be superfluous.

### SCARCITY OF NURSES AT WYNBERG HOSPITAL.

"M. F." writes from Wigan: The particulars of the nursing arrangements at the Wynberg Hospital, published in your pages, would greatly surprise the many trained nurses here, whose proffered services have been refused. Surely while the country rings with appeals to provide more men, and comforts for "Tommy" in action, something more ought to be done for him when he is wounded and in hospital. Is it fair to the sisters who are already out in South Africa to expect them to nurse efficiently at the rate of seven sisters to 600 patients? I have no wish to reflect upon the orderlies, who are useful if not indispensable. We send out eminent surgeons, then why hinder their work by false economy in an insufficient number of fully-trained nurses to carry out their instructions? Imagine, too, the wounded travelling 591 miles by rail! Surely if Florence Nightingale could minister "on the field," we who are endeavouring to follow her noble example can venture a little nearer to the scene of action than at the Case Hospital 591 miles away?

### NURSING AT HERBERT HOSPITAL, WOOLWICH.

A "SISTER-IN-CHARGE, A.N.S.R.," writes: I should like to give a few facts concerning military nursing as I find it. First as regards the sisters' duties in the wards. We have infinitely more to do than giving medicines and taking temperatures, as our rules make us responsible for the personal cleanliness of our patients, also that all diets and extras are supplied to them as ordered, and for the training of the orderlies in the methods of handling patients, applying dressings and preventing bed sores. In my regulations for the Army Nursing Service there is no rule as to the length of time to stay in the wards provided we are attending to our patients. As regards the matron or head, permit me to state that though there are only two of us, I was appointed senior sister when we entered on our duties here, and am responsible not only for the nursing, but also for the housekeeping and messing, so I think there must be an error in that statement. As your "Valued Correspondent" infers that the sisters adhere so rigidly to their rules, they must have omitted to read "Discipline and Duty," paragraph 214 of their regulations.

### UNTRAINED NURSES.

MISS BESSIE CAWOOD, 60, Talbot Road, Bayswater, writes: Would not the establishment of an examining board, in touch with the training schools of the country, be a great boon to the nursing profession? While it would not tend to substitute mere knowledge for training—as only nurses holding a training school certificate would be eligible for its examinations—it would at least insure that the nurses holding its certificate possessed a certain amount of necessary knowledge and education. It would tend—as the L.O.S. and Medico-Psychological have tended—to define and raise the standard of teaching and to secure something like uniformity. The examination could be made thoroughly practical and technical, a real test of the reality of a nurse's training, and for which it would be impossible to cram. The nervous woman who loses her head at an examination would do the same at any other crisis, and is not fit to be a nurse. The possession of that presence of mind which consists in remembering and doing what has previously been studied, at the right time, is the great distinction between a trained and an untrained nature. I know that it is impossible for an ignorant, incapable woman to pass through some of our training schools; but, speaking from real experience, I do not think that a three years' certificate guarantees a really trained nurse. Is it not a fact that the examinations in many schools are the merest farces, and have no bearing on the certificates given at the end of the training? A nurse, like a doctor, should not be considered qualified because she has spent a certain time in hospital. The grievance, dwelt on so frequently in your columns, that untrained women are sent out by institutions and paid for by the public, could not exist were it not that there is often little real distinction between the work of some women who have been three years and some who have been three months in a hospital. As the average nurse pays less for learning her profession than any other class of educated women workers, there should be no difficulty about fees. Most nurses would be thankful to pay for an opportunity of proving their efficiency and obtaining a certificate which would define their position as general nurses, in the same way that the L.O.S. certificate now defines the position of midwives.

### EASY SWINDLES.

"E. N. W.," writing from a town in the Midlands, says: Seeing the communications in your columns on this subject, I will tell you my experience relating (I feel sure) to the same impostor. On October 2nd I had a visit from a lady about one o'clock saying that she wished to find a home for the winter for her mother, who had hitherto lived with a son, now about to be married, and that she did not wish to make other permanent arrangements till the spring. A nurse-companion was to come with her, and they wished for a private sitting-room as well. I showed her the rooms, and she thought them rather small, as her mother was accustomed to a very large bedroom, and she finally agreed to take them, and she also agreed to my terms without any demur. She said that she wanted to come herself for six or eight weeks, as the doctors had ordered her a rest from housekeeping, and she wished them all to come the following Thursday, but I had not sufficient rooms disengaged, so could not receive her till the next week. She said she had travelled from Darlington that morning, and was to return at night, but wanted to make some purchases at Manchester on her way, only she had left her purse with a large amount of money in her husband's brief bag (he was a lawyer, she stated), and had only a few shillings with her. However, happily I was cautious, and did not lend her a farthing, though she stuck to me like a leech till the train departed for Manchester at 3.30 p.m. I had an appointment at two which I was obliged to keep. She waited for me outside, and then asked me to take her to the post office, where she despatched a telegram (for which she paid), and then invited me to a confectioner's, where we both had tea (she again paying). At the post office she wrote her name and address on a piece of paper, which she gave me, "Mrs. Cartwright, The Cedars, Darlington." I felt suspicious at her hint of wishing to borrow money from me, but otherwise I was completely taken in by her, as she spoke of people whom I knew well as if she also knew them well. I wrote to her the next day at the above address to say definitely which day I could receive her, but had my letter returned from the Dead Letter Office, and on consulting a law list found there was no lawyer of that name in Darlington. She was short and stout, with a Japanese cast of countenance, wore spectacles, was dressed in grey blue cloth coat and skirt, trimmed with handsome braid, and a small boat hat. She carried a cloak and a small bag, and talked like a lady.

### For Reading to the Sick.

Come unto me all ye that are weary and heavy laden and will give you rest. Take my yoke upon you, for I am meek and lowly in heart; and ye shall find rest unto your souls."—*St. Matt.* xi. 28.

Lord, a whole long day of pain
Now at last is o'er!
Ah, how much we can sustain
I have felt once more;
Felt how frail are all our powers,
And how weak our trust;
If Thou help not, these dark hours
Crush us to the dust.
Could I face the coming night
If Thou wert not near?
Nay, without Thy love and might
I must sink with fear:
Round me falls the evening gloom,
Sights and sounds all cease,
But within this narrow room
Night will bring no peace.
Come then, Jesus, o'er me bend,
Give me strength to cope
With my pains, and gently send
Thoughts of peace and hope.
—*From Lyra Germanica.*

**Reading.**

Cast all thy care on God. See that all thy cares be such as thou canst cast on God, and then hold none back. Never brood over thyself; never stop short in thyself; but cast thy whole self, even this very care which distresseth thee, upon God. Be not anxious about little things, if thou wouldst learn to trust God with thine all. Act upon faith in little things; commit thy daily cares and anxieties to Him; and He will strengthen thy faith for any greater trials. Rather, give thy whole self into God's hands, and so trust Him to take care of thee in all lesser things, as being His, for His own sake, whose thou art.—*E. B. Pusey.*

God beholds thee individually, whoever thou art. "He calls thee by thy name." He sees thee, and understands thee. He knows what is in thee, all thy own peculiar feelings and thoughts, thy dispositions and likings, thy strength and thy weakness. He views thee in thy day of rejoicing and thy day of sorrow. He sympathises in thy hopes and in thy temptations; He interests himself in all thy anxieties and thy remembrances, in all the risings and fallings of thy spirit. He compasses thee round, and bears thee in His arms; He takes thee up and sets thee down. Thou dost not love thyself better than He loves thee. Thou canst not shrink from pain more than He dislikes thy bearing it; and if He puts it on thee, it is as thou wilt put it on thyself, if thou art wise, for a greater good afterwards.—*J. H. Newman.*

Thou knowest all the present—each temptation,
Each toilsome duty, each foreboding fear;
All to myself assigned of tribulation,
Or to beloved ones, than self more dear!
All pensive memories, as I journey on,
Longings for vanished smiles and voices gone!

Thou knowest all the future—gleams of gladness,
By stormy clouds too quickly overcast—
Hours of sweet fellowship, and parting sadness,
And the dark river to be crossed at last.
Oh, what could confidence and hope afford
To tread that path, but this—Thou knowest, Lord!

Thou knowest, not alone as God, all-knowing—
As man, our mortal weakness Thou hast proved;
On earth, with purest sympathies o'erflowing,
Oh, Saviour! Thou hast wept, and Thou hast loved!
And love and sorrow still to Thee may come,
And find a hiding-place, a rest, a home.—*By H. L. L.*

### Notes and Queries.

**The contents of the Editor's Letter-box have now reached such unwieldy proportions that it has become necessary to establish a hard and fast rule regarding Answers to Correspondents. In future, all questions requiring replies will continue to be answered in this column without any fee. If an answer is required by letter, a fee of half-a-crown must be enclosed with the note containing the enquiry. We are always pleased to help our numerous correspondents to the fullest extent, and we can trust them to sympathise in the overwhelming amount of writing which makes the new rules a necessity.**

**Every communication must be accompanied by the writer's name and address, otherwise it will receive no attention.**

*Anti-Alcoholic Serum.*

(170) Would the editor please tell inquirer if it is true that a serum has been discovered which gives a distaste to drink, and to whom could she write to about it?

The reported discovery of an anti-alcoholic serum has still to be confirmed. Even if it be true, some time must elapse before it can be available for the general public. Inquirer should give her full name as well as address.

*Toxteth Infirmary.*

(171) I shall be much obliged if you will kindly tell me whether the Toxteth Infirmary, Liverpool, is a registered training school for nurses, and if its certificate would qualify the holder for a post in any other branch of the profession besides union infirmary nursing?—*E. M. H.*

One great difficulty in answering such questions as the above is that there is no register of training schools for nursing. We have no particulars as regards the nursing at Toxteth Infirmary, Liverpool, and should advise you to write to the Local Government Board, Whitehall, S.W.

*Training.*

(172) Could you tell me if a young lady would be able to take a situation in a general infirmary as a certificated nurse after serving three years as a probationer in a union infirmary, and getting certificates as nurse from there?—*B. McP.*

Yes, if the union infirmary fulfilled the requirements of the Local Government Board—that is, if it be a training school for nurses, and maintains a resident medical officer.

*Floors.*

(173) Can you tell me what would whiten a pitch pine floor in hospital wards?—*Nurse E.*

The floors may be whitened by planing, for which a carpenter must be employed; by oxalic acid, dangerous to use; or by scrubbing with soda and chloride of lime. A good result was obtained on a very bad floor by the following means. It was scrubbed free from grease, a thick paste of chloride of lime was spread over it, and the sun and air freely admitted. As the paste dried it was watered with a watering can. When this treatment had lasted a couple of days the boards were scrubbed and dried clean. A floor may be kept white by adding a handful of whiting to the scrubbing water.

*South Africa.*

(174) I am most anxious to go out to South Africa to assist in the nursing of our troops there. Will you kindly tell me to whom I ought to apply?—*Nurse P.*

The Army Nursing Reserve, 18, Victoria Street, Westminster.

*Sanatorium, N.B.*

(175) Will you kindly let me know of any hospital or home in Scotland where a nurse may be treated under open-air treatment for consumption at a moderate fee?—*M. R. P.*

The "West London Medical Journal" for October gives the Victoria Hospital for Consumption, Craigleith, Edinburgh (Dr. A. V. Philip).

*Training.*

(176) Would you kindly tell me which would be considered the best, a two years' provincial and a one year's London training, or a three years' provincial training?—*Probationer.*

"Probationer" should read our rules to correspondents. Secure a three years' certificate, and supplement it with the best experience obtainable.

*Waltham Training School for Nurses.*

(177) I was much interested in your report. As I am desirous of having my daughter trained (whose age is 19) as a hospital nurse, may I ask you to be kind enough to give what information you can of this school, and if she can be placed there without fees of any kind and without difficulty?—*Anxious.*

Write to the Principal of the Waltham Training School for Nurses, 714, Main Street, Waltham, Mass., U.S.

*Homes for Gentlewomen.*

(178) Can you kindly give me addresses of small flats for gentlewomen in London (such as the Campden Houses), or tell me to whom I could apply for particulars?—*Sister C.*

You will find a very complete list in "The Englishwoman's Year Book" (Adam and Charles Black), price 2s. 6d.

*Standard Books of Reference.*

"The Nursing Profession: How and Where to Train.' 2s. net.

"The Nurses' Dictionary of Medical Terms." 2s. 6d. net.

"Burdett's Series of Nursing Text-Books." 1s. each.

"A Handbook for Nurses." (Illustrated.) 5s.

"Nursing: Its Theory and Practice." New Edition. 3s. 6d.

"Helps in Sickness and to Health." Fifteenth Thousand. 5s.

All these are published by The Scientific Press, Ltd., and may be obtained through any bookseller or direct from the publishers, 28 & 29, Southampton Street, London, W.C.

